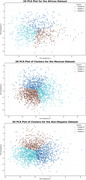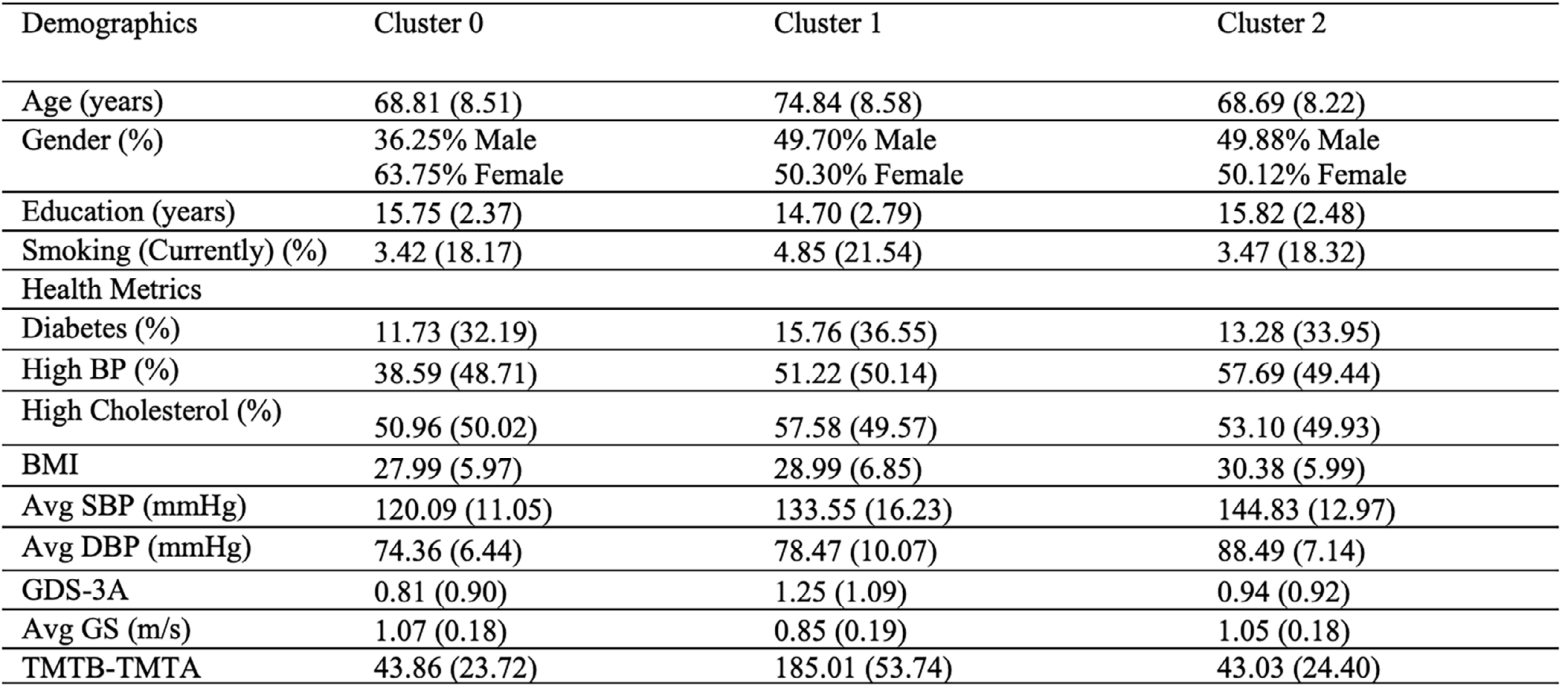# The AGED Phenotype: A vascular phenotype of cognitive impairment across diverse populations

**DOI:** 10.1002/alz70860_107760

**Published:** 2025-12-23

**Authors:** Adrian Noriega de la Colina, Nila Z. N. Jan, Caitlin S. Walker, Arthur F. Kramer, Maiya R. Geddes

**Affiliations:** ^1^ McGill University, Montreal, QC, Canada; ^2^ Montreal Neurological Institute‐Hospital (The Neuro), McGill University, Montreal, QC, Canada; ^3^ Khalifa University, Abu Dhabi, Abu Dhabi, United Arab Emirates; ^4^ Montreal Neurological Institute‐Hospital Cognitive Neuroscience Research Group, McGill University, Montreal, QC, Canada; ^5^ Beckman Institute, University of Illinois, Urbana, IL, USA; ^6^ Center for Cognitive & Brain Health, Northeastern University, Boston, MA, USA; ^7^ McGill University Research Centre for Studies in Aging, McGill University, Montreal, QC, Canada; ^8^ StoP‐AD Centre, Douglas Mental Health Institute Research Centre, Montreal, QC, Canada; ^9^ The Neuro, Faculty of Medicine, McGill University, Montreal, QC, Canada; ^10^ Centre for Studies in the Prevention of Alzheimer's Disease, Douglas Mental Health Institute, McGill University, Montreal, QC, Canada

## Abstract

**Background:**

The AGED phenotype—Apathy, Gait Disturbance, and Executive Dysfunction—has been proposed as an early marker of vascular cognitive impairment. This study examines its prevalence and cross‐ethnic validity, exploring associations with vascular risk factors and cognitive decline. Identifying syndromic markers such as AGED may improve early intervention strategies and risk stratification for cognitive aging, particularly in underrepresented populations.

**Method:**

Data were drawn from two discovery cohorts—the Daily Activity Study of Health (DASH; *n* = 37) and the Healthy Aging Brain Study (HABS; *n* = 52)—and externally validated in The Health & Aging Brain Study ‐ Health Disparities (HABS‐HD; *n* = 4,477). The HABS‐HD cohort comprised African American (*n* = 737), Mexican American (*n* = 1,831), and Non‐Hispanic White (*n* = 1,909) adults (Mean age=66.00, SD=8.4).

The AGED phenotype was defined using apathy (GDS‐3A), gait speed (4‐meter test), and executive function (Trail Making Test B, TMT‐B). K‐means clustering (k=3) identified low, intermediate, and high‐risk AGED phenotypes. Vascular risk factors—including blood pressure, diabetes, BMI, and cholesterol—were analyzed across clusters. Clustering stability was assessed via Jaccard Index, and principal component analysis (PCA) aided dimensionality reduction.

**Result:**

Three AGED clusters emerged in DASH and HABS, successfully replicated in HABS‐HD:

1. Low‐risk (50.1%)—faster gait (>1.0 m/s), preserved cognition, low apathy.

2. Intermediate‐risk (34.8%)—moderate gait (0.8–1.0 m/s), mild executive dysfunction, moderate apathy.

3. High‐risk (15.1%)—slow gait (<0.8 m/s), severe executive dysfunction, high apathy, linked to higher systolic BP (≥135 mmHg), diabetes, and cognitive impairment (TMT‐B >200s).

Prevalence of the high‐risk AGED phenotype:

• African Americans (17.3%)—higher BP (151.07 mmHg), greater executive dysfunction (TMT‐B=244.45s).

• Mexican Americans (21.6%)—highest prevalence, lower education (7.06 years), high diabetes (45.21%).

• Non‐Hispanic Whites (11.8%)—better gait (1.08 m/s) but higher hypertension (59.57%).

Jaccard Index demonstrated high clustering stability (DASH=0.901, HABS=0.925, HABS‐HD=0.915–0.940).

**Conclusion:**

The AGED phenotype represents a vascular variant of cognitive impairment, with higher prevalence in Mexican Americans and African Americans, supporting an ethnic risk gradient. External validation confirms its generalizability. Given its vascular associations, AGED may serve as a preclinical marker for early intervention in cognitive decline. Future studies should assess its predictive validity for dementia and targeted interventions.